# Healthy Brain Initiative and Building Our Largest Dementia Infrastructure initiatives: 20 years of building a strong public health infrastructure

**DOI:** 10.1093/geront/gnaf225

**Published:** 2025-10-03

**Authors:** Lisa C McGuire, Heidi L Holt

**Affiliations:** Gerontological Society of America, Strategic Alliances & Practice Innovation, Washington, District of Columbia, United States; Centers for Disease Control and Prevention, Alzheimer’s Disease Program, Atlanta, Georgia, United States

The year 2025 marks both the 20th anniversary, and an uncertain future for, the Centers for Disease Control and Prevention’s (CDC) Healthy Brain Initiative (HBI), a national effort to address Alzheimer’s disease and related dementias (ADRD) as critical public health issues. Launched in 2005 and expanded by the 2018 Building Our Largest Dementia (BOLD) Infrastructure for Alzheimer’s Act, the HBI significantly advanced public health capacity in dementia risk reduction, early detection, caregiving support, and national surveillance. Through the HBI Road Map Series, the Healthy Brain Research Network (HBRN), and initiatives targeting high-risk populations, the HBI integrated brain health into national policy and public health infrastructure. Core to these efforts were the Behavioral Risk Factor Surveillance System (BRFSS) and the National Health and Nutrition Examination Survey (NHANES), which provided essential data on subjective cognitive decline (SCD) and caregiving.

The HBI was launched out of a recognition that ADRD were growing public health issues that were not fully recognized by the public health community, with nearly 5 million adults 65 years old and older in the United States living with Alzheimer’s dementia in 2005. The US Congress in 2005 recognized the public health impact of Alzheimer’s disease with its effect on public and private health care systems, community services and supports, and people living with Alzheimer’s and their families, by initiating support to establish the HBI at the CDC. In 2020, the US Congress further solidified its financial support for public health action for Alzheimer’s by establishing the BOLD Infrastructure program, establishing nationwide expertise, and developing an infrastructure to improve the public health response to ADRD to uniformly and comprehensively implement the CDC’s HBI Road Map Series across the US.

Through the HBI and BOLD, the CDC, in collaboration with federal, state, local, territorial, tribal, and non-governmental partners, made extraordinary strides in establishing ADRD nationally as a public health issue and providing support to public health departments to establish and implement a response within their jurisdictions. The number of people living with Alzheimer’s dementia continues to grow from an estimated 5 million in 2015 to an estimated over 7 million in 2025. By 2060, that number will approach 14 million (Alzheimer’s Association, 2025), making these collaborative efforts even more critical with research, clinical care, direct services, and public health all collaborating to improve dementia risk reduction, early detection and diagnosis, and care. This article will explore and celebrate the 20-year accomplishments of the HBI, and the 5-year accomplishments of BOLD as of the finalization of this article in September 2025, as shown in Figure 1.

**Figure 1.**
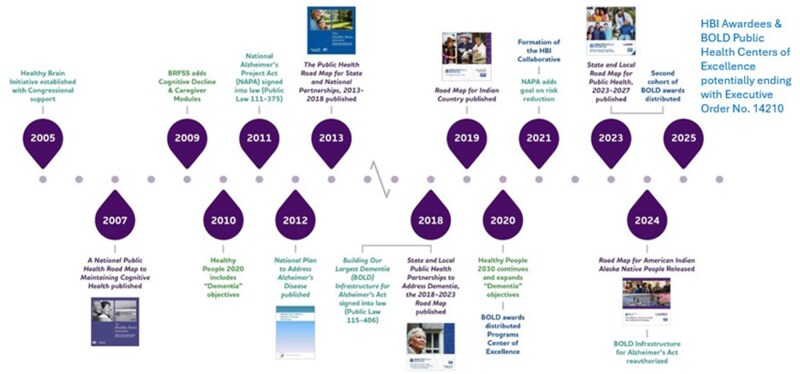
Twenty years of National Public Health ADRD initiatives.

## HBI: Public health’s path

Over the 20 years of HBI, the role of public health to address brain health and ADRD was established. Nationally, the HBI has put ADRD and brain health on the public health radar. The HBI not only made the case that ADRD is a public health issue, but it also demonstrated that public health organizations—both public and private—have significant opportunities to contribute to ongoing efforts and introduce innovative approaches in ADRD, utilizing the strengths of public health. The public health sector, consistent with the Essential Public Health Services (Public Health National Center for Innovations & de Beaumont Foundation, 2020), excels at enhancing the capabilities of public health agencies, utilizing partnerships effectively, improving data use and collection, and exploring new methods for incorporating ADRD and brain health into existing public health strategies.

Before 2005 and HBI, federal efforts to address Alzheimer’s disease mainly focused on research and services to understand the disease process and provide services and support to persons living with the disease and their caregivers. The US Congress recognized the gap between emerging research and the care of those affected by ADRD that could be bridged by utilizing and enhancing the capabilities of public health agencies. With appropriations and authorization from Congress, the CDC established the HBI in 2005. This charged the CDC to take on the topics of brain health and ADRD for the first time, leading a national initiative for public health. This action initiated the longstanding collaboration between the Alzheimer’s Association (2005–2025) and the CDC to advance understanding of and support for brain health, including ADRD as a central part of public health practice.

The HBRN, a thematic network within the CDC Prevention Research Centers program, was a collaborative initiative established in 2014 by the CDC to address the growing public health challenges associated with cognitive health and aging. Coordinated by the University of Washington Health Promotion Research Center, the network was built upon the expertise and collaborative strengths comprised of six academic centers–University of Washington, Oregon Health & Science University, University of Arizona, University of Illinois at Chicago, University of Pennsylvania, and the University of South Carolina—and two affiliate centers—University of Houston and University of Pittsburgh (Centers for Disease Control and Prevention, 2017). Each center contributed to the overarching goals of promoting brain health, addressing cognitive impairment, and supporting caregivers. During its operation from 2014 to 2019, the HBRN led and engaged experts from across the country in developing a consensus-based research agenda related to public health in cognitive health, cognitive aging, and cognitive impairment. The network evaluated public messaging around promoting early detection of dementia in diverse populations, including African American, Asian American, Latino, and LGBTQ communities. Research indicated that culturally relevant messages have the potential to increase early detection of ADRD (University of Washington, nd). The HBRN also focused on building a strong evidence base for communication and programmatic interventions to improve or maintain cognitive function. This included translating that evidence base into effective public health programs and practices in states and communities (Arizona Center on Aging, nd). Although the HBRN concluded its formal activities in 2019, its legacy includes a cadre of scholars and researchers from many disciplines, including public health, medicine, and behavioral health, an expanded network of community organizations and academic centers, scholarly publications, and research-based resources that can continue to support communities’ efforts toward better cognitive health. The network's scholarly publications, research-based resources, and community partnerships remain valuable assets for ongoing efforts to promote cognitive health and support individuals affected by cognitive impairment.

CDC expanded its partnership to establish The National Brain Health Center for African Americans (NBHCAA) with the Balm in Gilead (2015–2020; The Balm in Gilead, nd). This partnership with the NBHCAA was instrumental in raising awareness of brain health issues among African Americans by engaging faith-based communities, health care providers, and community leaders to enhance awareness, education, and support for brain health within these communities. The NBHCAA established Memory Sunday as the flagship program of the NBHCAA, designed to raise awareness about Alzheimer’s disease and other dementias within African American communities. Held annually, this initiative encourages congregations to dedicate a Sunday service to discussing cognitive health, providing educational materials, and supporting individuals affected by these conditions. The NBHCAA offers a comprehensive resource library that includes resources about ADRD and how to manage it; information on the impacts of stroke, epilepsy, and traumatic brain injury; and materials for caregivers and families navigating brain health challenges. The NBHCAA plays a role in addressing the cognitive health challenges faced by African American communities. Through its programs and partnerships, the NBHCAA strives to reduce health disparities, promote awareness, and provide support for individuals and families affected by brain health issues.

To further build the public health infrastructure of ADRD, the HBI made awards to 3 organizations (2020–2025) serving populations at highest risk of dementia with a national reach (Centers for Disease Control and Prevention, 2024a). These organizations were: The International Association for Indigenous Aging focused on American Indian and Alaska Native (AI/AN) adults, UsAgainstAlzheimer’s focused on African American and Hispanic/Latino adults, and the University of Illinois Chicago focused on persons with intellectual and developmental disabilities (IDD). Each of these organizations engaged with community and state, local, territorial, and tribal partners on public health policies and programs; translated research into public health practice; and developed training and communication products to improve population health outcomes of those most at risk for ADRD and caregivers of those with dementia. Within this issue, the work of each organization will be highlighted: (1) Donnellan, Eppes, Dorame et al. describe in their paper that tailoring brain health and dementia information with and for Native communities is vital for enhancing awareness and fostering culturally relevant interventions; (2) Delgado and Monroe discuss the importance of personalizing messages for African American and Hispanic/Latino audiences in such a way to be culturally relevant to and resonate with minoritized communities and that those messages be delivered by trusted members of the community, such as known health care providers, social workers, and community health workers; and (3) Marks, Sisirak, Janicki et al. address key aspects of brain health and dementia-capable care for individuals with IDD, proposing a culturally responsive framework that integrates brain health promotion with dementia-capable services while considering disability intersectionality. Unfortunately, the future of these awards is uncertain, due to the restructuring of the federal government.

### Evolution of the HBI Road Map Series

The crown jewel of the HBI suite of products is the HBI Road Map Series, each edition collaboratively developed by the CDC and the Alzheimer’s Association. Each Road Map within this series established a timely set of national priorities and actions that public health could implement to fully integrate attention brain health, ADRD, and caregiving into public health practice. To ensure that the HBI Road Map remains current and reflects the latest advances in science, policy, and programmatic activity, the Road Maps are updated at least every 5 years by an extensive group of relevant subject matter experts, professional organizations, and public health professionals. Since the publication of the initial HBI Road Map, the series now comprises six editions: Four designed for State and Local Public Health, and two for Tribal Communities, as shown in Figure 1.

The initial HBI Road Map, *The National Public Health Road Map to Maintaining Cognitive Health*, was published in 2007 (Centers for Disease Control and Prevention and the Alzheimer’s Association, 2007). This was the first document to establish national public health priorities needed to address ADRD and promote brain health nationally and served as the pioneering framework for addressing dementia as a public health priority. The development of this document included over 200 subject matter experts in the fields of brain health, cognition, and ADRD; national organizations including the American Association of Retired Persons (now AARP) and the National Association of Chronic Disease Directors; and federal agencies including the Administration on Aging and National Institute on Aging. This first Road Map and its 44 actions were aimed at enhancing public awareness, improving surveillance, and fostering research into cognitive health. The actions were organized into 8 main clusters: Disseminating information, translating knowledge, conducting surveillance, implementing policy, measuring cognitive impairment and burden, moving research into practice, conducting intervention research, and developing capacity.

Following the 2007 Road Map, there was increasing attention and national action on ADRD, including the passage of the National Alzheimer’s Project Act in 2011 (see Figure 1). Building upon the 2007 document and reflecting updates to the national ADRD landscape, the second edition of the HBI Road Map Series was released in 2013 by CDC and the Alzheimer’s Association. The 2013 Road Map, *The Public Health Road Map for State and National Partnerships, 2013–2018* (Alzheimer’s Association and Centers for Disease Control and Prevention, 2013), emphasized the role of state and local public health departments in addressing dementia. This edition focused on state- and local-level actions designed for state and local public health departments to implement that anticipate and adapt to innovations and developments in Alzheimer’s research and best practices in programmatic delivery. The development of this Road Map reflected the efforts of hundreds of experts from national, state, local, territorial and tribal governments, non-governmental organizations, and subject matter expertise from academic institutions. The 2013 Road Map emphasized the importance of using public health resources to enhance brain and cognitive function, understand and address cognitive impairment, and better support caregivers. This document outlined 35 actions, including: developing public health surveillance systems for cognitive decline and caregiving, translating research findings into public health practice, creating effective public health initiatives, and fostering collaborations between public health and aging services professionals. It introduced strategies to integrate dementia care into existing public health infrastructures and highlighted the importance of community-based interventions.

In 2018, the CDC and the Alzheimer’s Association released the third HBI Road Map in the Series, *State and Local Public Health Partnerships to Address Dementia, The 2018–2023 Road Map* (Alzheimer’s Association and Centers for Disease Control and Prevention, 2018). There were 25 specific actions to address dementia in state, local, and territorial communities that were aligned with the Essential Public Health Services framework (Public Health National Center for Innovations & de Beaumont Foundation, 2020). This alignment ensured that ADRD-related initiatives were seamlessly incorporated into broader public health efforts, making them consistent with the way public health professionals and departments operate, promoting sustainability and systemic change. The 2018 Road Map continued to guide state, local, and territorial public health departments and their partners to act through policy, systems, and environmental changes. Highlighting the need for continued attention to the issue, the actions within the 2018 Road Map provided a solid foundation for public health to continue building a robust infrastructure along with strong and diverse partnerships, and to integrate the actions into ongoing public health efforts.

Recognizing the unique challenges faced by AI/AN populations, tribal health departments, and communities–including tribal sovereignty and variable public health infrastructures–CDC and the Alzheimer’s Association in 2019, released the first-ever public health guide tailored for leaders within AI/AN communities to develop a response to ADRD. The *Healthy Brain Initiative Road Map for Indian Country* (Alzheimer’s Association and Centers for Disease Control and Prevention, 2019) was created and reviewed by a diverse group of public health organizations, tribal organizations, and AI/AN subject matter experts. Community listening sessions were conducted within AI/AN communities to listen to their needs and provide input into the document. The resulting culturally-tailored guide helped tribes, nations, pueblos, bands, villages, and urban Indian organizations lay the public health foundation to improve brain health, address dementia, and better meet the needs of caregivers.

The fifth edition of the HBI Road Map Series is the fourth version for state and local public health, *Healthy Brain Initiative: State and Local Road Map for Public Health, 2023-2027* (Alzheimer’s Association and Centers for Disease Control and Prevention, 2023) which was released in 2023 by CDC and the Alzheimer’s Association. This version expands on previous progress, places a stronger emphasis on health equity, partnerships, and data utilization, and addresses the social factors that impact brain health. It introduces 24 specific actions designed to help public health professionals lead with urgency and act for impact in their communities to improve brain health across the life course and support caregivers, with specific emphasis on reducing the risk of cognitive decline, supporting caregivers, and enhancing early detection and diagnosis. In the development of this Road Map, input and guidance from over 100 experts were incorporated and included a greater emphasis on partnerships and addressing populations at high risk of developing ADRD. This Road Map centers on public health as a way for leaders, scientists, health and social care providers, public health professionals, and communities to connect for maximum action and impact, with the overarching vision that everyone deserves a life with the healthiest brain possible.

*Healthy Brain Initiative Road Map for American Indian and Alaska Native Peoples (* Alzheimer’s Association and Centers for Disease Control and Prevention, 2024) is the second version developed for tribal communities, which was released in 2024 by the CDC and the Alzheimer’s Association. It is the sixth and potentially the final edition of the HBI Road Map Series. This Road Map builds on the progress and momentum of the 2019 *HBI Road Map for Indian Country* and advances the vision that everyone deserves a life with the healthiest brain possible. This updated edition was developed with respect for the diverse history, cultures, traditions, and practices of AI/AN peoples across the country. This 2024 Road Map focuses on health equity through a strengths-based approach, drawing on the traditional practices and cultural activities that have kept AI/AN communities healthy for thousands of years. Included are examples of how AI/AN communities used the 2019 Road Map to advance brain health and support AI/AN Elders living with ADRD. This Road Map’s development was led by a Leadership Committee of tribal leaders, physicians, subject matter experts, and researchers in public health and across the care continuum, reflecting input from over 200 community members and professionals working in AI/AN communities.

The article by Fadel, Shean, Jackson et al. describes how the HBI Road Map Series has been used as a launching point to support the integration of ADRD in health departments nationwide. It highlights how the Alzheimer’s Association coordinates the implementation of each Road Map through strategic engagement with health departments at the state, local, territorial, and tribal level. Participants from a growing number of HBI partners work together to implement public health strategies that promote brain health, address dementia, and support people living with dementia and their caregivers. Recognizing opportunities to influence the trajectory of public health action, the Alzheimer’s Association prioritizes growing the availability and use of dementia-related public health data and equipping the public health workforce with the knowledge and confidence to make change.

### Building and utilizing a national surveillance system

Public health surveillance is the systematic and continuous collection, analysis, and interpretation of health-related data. It is a cornerstone of public health practice, helping to understand the magnitude of a disease or condition, identify trends and risk factors, and assess the effectiveness of public health interventions. The application of this data can inform policies, guide interventions, and monitor progress across the United States. Healthy Brain Initiative led the charge to establish national surveillance on a precursor of ADRD, subjective cognitive decline (SCD), and caregiving.

Given the foundational importance of data to public health, aspects of data collection and utilization have been central to each edition of the HBI Road Map Series. CDC developed and implemented robust surveillance efforts to collect public health data on cognitive decline and caregiving. A key component of the HBI's surveillance efforts is the BRFSS (Centers for Disease Control and Prevention, 2025) that includes regularly updated modules on SCD and caregiving. BRFSS is the nation’s premier system of health-related telephone surveys that collect state data about US residents regarding their health-related risk behaviors, chronic health conditions, and use of preventive services. BRFSS collects data in all 50 states as well as the District of Columbia and three U.S. territories. BRFSS completes more than 400,000 adult interviews each year, making it the largest continuously conducted health survey system in the world. Population-based measures of SCD and caregiving, such as those collected through the BRFSS, can be extremely useful to the public health community, health care providers, researchers, and policymakers in informing decision-making.

In 2007, CDC, with the assistance of national experts, developed a 10-question module within BRFSS to measure cognitive impairment, along with its associated effects. This module was developed in response to recommendations in the inaugural HBI Road Map, which emphasized the need for population-based surveillance of cognitive health. Further enhancing its commitment to cognitive health surveillance, in 2009, the CDC refined this module to measure SCD and its associated effects. This module was first pilot tested by five states in 2009, followed by 47 states and territories in 2011, 2012, and 2013 as states added questions on their BRFSS. The CDC consulted with data users and convened a panel of experts on surveillance, survey development, and cognition to revise the module based on feedback and advances in understanding SCD, which are self-reported difficulties in thinking or memory. This revised module, now called the Cognitive Decline Module, was included as an official Optional Module in the beginning of the 2015 BRFSS with six questions. In 2023, a further revised 5-question module was released.

In 2009, state health departments administered the first Caregiver Module in their annual BRFSS survey as states added questions. This module was developed to capture information aspects of the caregiving situation, including intensity, duration, and type of assistance provided, as well as aspects of the caregiver’s physical and mental health. These data are instrumental in capturing an accurate picture, both in states and nationwide, of caregivers providing care or assistance to a family member or friend due to disability, disease, or other health problems. This module was updated in 2016, 2019, and 2024 to refine the questions and ensure the data collected are current with the latest research and public health practice.

Both the BRFSS Caregiver and Cognitive Decline Modules are routinely administered by state and territorial health departments across the US, and analysis of the resulting data informs programs, science, and policy. All 50 states, Puerto Rico, and the District of Columbia have administered these modules, which provide data for action and decision-making at both the state and national levels. This data is instrumental in supporting ongoing health and public health projects, informing decision-making and priority setting, and monitoring trends across time. Subsequent editions of the HBI Road Map Series have continued to advocate for the integration of BRFSS modules to collect data on cognitive decline and caregiving. For instance, the 2023–2027 Road Map recommended implementing the BRFSS optional modules for Cognitive Decline and Caregiving and using the data to develop and inform programs and policies (Alzheimer’s Association and Centers for Disease Control and Prevention, 2023).

The information gained from these two optional BRFSS Modules benefits the public health community, health and social-care providers, researchers, and policymakers. Population measures derived from these modules enable researchers to better estimate populations that may be more at risk for cognitive impairment based on their SCD status. The impact achieved using these data has been widespread. Both the CDC Alzheimer’s Disease and Healthy Aging Data Portal (Centers for Disease Control and Prevention, 2024b) and Chronic Disease Indicator (Centers for Disease Control and Prevention, 2024c) projects include information gathered from these Caregiver and Cognitive Decline BRFSS Modules, including the raw data and selected analyses, which are publicly accessible. Both databases are periodically updated upon the release of new data and recommendations from subject matter experts. Additionally, state-specific fact sheets utilizing BRFSS data on cognitive decline and dementia caregiving are publicly available (Alzheimer’s Association, nda).

Additionally, the CDC sponsored data collection through the NHANES, a biennial national survey of the US population’s health and nutrition status (Centers for Disease Control and Prevention, 2024d). It examines a nationally representative sample of about 5,000 people through interviews, physical exams, and laboratory tests. Data collection is completed by trained personnel operating out of a fleet of mobile examination centers. Data specifically on cognitive performance in adults 60 years and older was collected during several survey cycles (2011–2012, 2013–2014, and 2019–2020). NHANES is the only national data source that collects cognitive performance data alongside other biometric information gathered from physical exams with participants. The NHANES sample conducted in 2019–2020 was collected from 18 sites. Typically, NHANES includes 30 sites, but the 2019–2020 cycle was cut short in March 2020 due to the COVID-19 pandemic (Centers for Disease Control and Prevention, nd). For that reason, these survey data are considered a convenience sample and are not considered representative of the US population. Caregiving questions are included in the current cycle of NHANES.

## Infrastructure for Alzheimer’s Act

The BOLD Infrastructure for Alzheimer's Act (P.L. 115-406; U.S. Congress, 2018b), enacted in 2018, is a landmark federal initiative led by the CDC to enhance the US public health infrastructure in addressing ADRD (Centers for Disease Control and Prevention, 2024e). This legislation aims for uniform implementation of the HBI Road Map Series across the US, focusing on dementia risk reduction, early detection and diagnosis, prevention of avoidable hospitalizations, and support for dementia caregiving. The paper by Kaskie, Bobitt, Shah et al. included in this issue describes the process that built BOLD and key policy issues and implications.

BOLD authorized the CDC to fulfill the three components of legislation (Centers for Disease Control and Prevention, 2024e): First, to establish public health centers of excellence (PHCOE) that focus on specific areas such as dementia risk reduction, early detection, and caregiving; second, to provide funding to public health departments in state, local, territorial, and tribal jurisdictions to implement dementia-related public health strategies from the HBI Road Map Series; and third, to enhance data analysis and reporting by improving the collection and dissemination of data related to ADRD to inform public health actions.

As part of the BOLD Act, the CDC created three BOLD PHCOE in 2020 through 2025, each specializing in a critical area related to ADRD (Centers for Disease Control and Prevention, 2024e). The Alzheimer’s Association’s BOLD PHCOE on Dementia Risk Reduction (Alzheimer’s Association, ndb) led efforts to develop and disseminate strategies aimed at reducing the risk of dementia through lifestyle modifications and public health interventions. The paper by Roberts, Leis, Colcombe et al. describes how this PHCOE worked to build and increase public health capacity for risk reduction. The NYU Grossman School of Medicine’s BOLD PHCOE on Early Detection and Diagnosis (NYU Grossman School of Medicine, nd) focused on improving early detection practices, making dementia screening a routine part of health care to facilitate timely diagnosis and intervention and the paper by Chodosh, Borson, Nordyke et al highlights the innovations and challenges faced. The University of Minnesota’s (University of Minnesota, nd) BOLD PHCOE on Dementia Caregiving worked to support family caregivers by providing resources, training, and strategies to enhance caregiving capabilities and reduce caregiver burden. The BOLD PHCOE on Dementia Caregiving described their past work and potential future activities to elevate dementia caregiving as a priority for public health in the article by Gaugler, Johnson, Epstein-Lubow et al. The PHCOEs serve as a resource to the nation to identify, translate, and disseminate promising scientific research and best practices of these dementia topic areas, make resources available free of charge to public health professionals and other individuals looking to improve their community’s dementia response. These PHCOEs serve an essential role through a nationwide, systematic public health approach to increase the adoption of evidence-informed best practices by state, local, territorial, tribal, and other public health programs. Within this issue, their work will be highlighted. Unfortunately, these three BOLD PHCOEs are the first and potentially last cohort, as these awards are not being recompeted in 2025.

In 2020 and 2021 through 2023, the CDC established the first-ever BOLD Public Health Programs that provided support to 23 state, local, and tribal public health departments to implement actions in the HBI Road Map Series. This funding enabled public health departments to build their capacity to address dementia more comprehensively and develop, update, and implement jurisdiction-wide ADRD strategic plans.

The BOLD Public Health Programs were expanded in 2023 after receiving increased congressional Appropriations to support 43 state, local, territorial, and tribal public health programs (Centers for Disease Control and Prevention, 2024f). These 5-year awards focused on strengthening community partnerships and improving their public health response to dementia risk reduction, early detection and diagnosis, referral to services, and improved connections between clinical settings and aging services networks. The BOLD Public Health Programs also seek to enhance supports for caregivers of persons living with dementia and to integrate caregivers as an essential part of the health care team.

Since the initial BOLD Public Health Programs were established in 2020, recipients have created or strengthened 45 ADRD coalitions in 35 states, 7 counties and cities, the District of Columbia, Puerto Rico, and 1 tribal organization. These represent over 1,500 organizations dedicated to improving the public health response to dementia in their communities, most of which are doing so for the first time, by enhancing the public health infrastructure in their jurisdictions. The article by Shean, White, Felten et al. describes successes of several BOLD Programs, demonstrating how they enacted change in their jurisdictions to make a difference.

In December 2024, the BOLD Act was reauthorized by Congress through the BOLD Infrastructure for Alzheimer’s Reauthorization Act of 2024 (Public Law 118-142; U.S. Congress, 2024b). This bipartisan legislation reauthorized the BOLD Program through 2029, as shown in Figure 1. This charges the CDC to reauthorize programs focused on state, local, territorial, and tribal health departments, Alzheimer’s and Related Dementias PHCOE, and data collection on Alzheimer's and related dementias.

To facilitate this multi-component approach to fully integrate brain health and ADRD into public health practice, the HBI Awardees and BOLD PHCOE awardees formed the HBI Collaborative (see Figure 1; Alzheimer’s Association, ndc) to expand collective impact and use a research and data-driven approach to translate the latest science and increase public health focus for dementia action. The HBI Collaborative is co-chaired by the CDC and the Alzheimer’s Association, and its collaborative approach enhances national, state, local, territorial, and tribal public health knowledge and capacity, amplifying the progress being made in public health.

The BOLD Infrastructure for Alzheimer’s Act represents a comprehensive approach to addressing the growing challenge of dementia in the United States. By strengthening public health infrastructure, enhancing early detection, supporting caregivers, and promoting risk reduction, the BOLD Act aims to improve the quality of life for individuals affected by dementia and their families. Ongoing evaluation and adaptation of these initiatives will be essential to meet the evolving needs of the aging population.

## Key national initiatives paving the way toward progress

In the 20 years of HBI, there were additional landmark federal milestones or initiatives that contributed to the national ADRD landscape and have led to considerable impacts. National efforts to integrate brain health into ongoing planning and policy initiatives, include the addition of dementia-specific Healthy People objectives (Office of Disease Prevention and Health Promotion, 2022), The National Alzheimer's Project Act (NAPA; P.L. 111–375; U.S. Congress, 2011), and the Recognize, Assist, Include, Support, & Engage (RAISE) Family Caregivers Act (U.S. Congress, 2018a). These are the first-ever milestones that enabled national action, leveraged partnerships, and offered a pathway to collaborative action and growth to address these goals and to improve the health and well-being of people living with ADRD and their families.

For the first time, Dementia, including Alzheimer’s Disease (DIA), was included as a new objective in Healthy People 2020 (published in 2010; see Figure 1; Office of Disease Prevention and Health Promotion, 2022). Healthy People objectives are measurable health priorities for the nation designed to improve health and well-being. Two dementia-specific objectives were added to improve care and quality of life for individuals living with dementia, focusing on early diagnosis, reducing preventable hospitalizations, and addressing the needs of caregivers. The objectives were “Increase the proportion of older adults with dementia, or their caregivers, who know they have it (DIA-01)” and “Reduce the proportion of preventable hospitalizations in older adults with dementia (DIA-02).” The development of Healthy People 2030 prioritized a robust effort to update and reduce the number of key objectives (Office of Disease Prevention and Health Promotion, nd). Despite this priority, the number of DIA objectives increased from two to three, signifying the growing attention and public health capacity to address dementia at the population level. The third objective added to Healthy People 2030 (published in 2020; see Figure 1) is “Increase the proportion of adults with SCD who have discussed their symptoms with a provider (DIA-03).”

The National Alzheimer's Project Act (NAPA; see Figure 1) (P.L. 111-375; US Congress, 2011), signed into law on January 4, 2011, marked a transformative shift in the United States’ approach to ADRD (U.S. Department of Health and Human Services, 2024). NAPA has been instrumental in transforming the national response to Alzheimer's disease. It has led to significant increases in federal funding for Alzheimer's research, the development of public health infrastructure to support individuals with dementia, and improved access to quality care. Before NAPA, there was no coordinated national strategy to address the growing public health challenge posed by Alzheimer's disease. NAPA established a comprehensive framework to guide federal efforts in research, care, and services for individuals affected by dementia.

NAPA legislation mandated the creation of a National Plan to Address Alzheimer's Disease (National Plan) with the goal of both preventing future cases of ADRD, to better meet the needs of the millions of American families currently living with this disease, and to establish the public-private Advisory Council on Alzheimer's Research, Care, and Services (Advisory Council; U.S. Department of Health and Human Services, 2024). The National Plan, first published in 2012 and then annually, outlines strategic approaches to the original five ambitious goals: Prevent and effectively treat Alzheimer’s disease by 2025; enhance care quality and efficiency; expand support for people with Alzheimer’s disease and their families; enhance public awareness and engagement; and improve data to track progress. The Advisory Council, established in 2011, oversees the implementation of the National Plan, provides recommendations to the US Department of Health and Human Services (HHS) and Congress on priority actions to expand, coordinate, or condense programs, and evaluates the nation's progress in preparing for the escalating burden of ADRD. The Advisory Council includes members representing several federal agencies specified in the legislation who work on dementia, including the CDC, and several members from outside the federal government, who provide their expertise to help the HHS Secretary continue to advance our understanding of how to prevent, detect, and more effectively ­manage Alzheimer’s.

The NAPA Advisory Council formed an ad hoc Risk Reduction subcommittee to recommend an additional goal for inclusion in the national plan that addressed strategies for mitigating risk factors associated with ADRD in July 2020, based on the growing body of scientific evidence regarding risk factors for ADRD (Gore et al., 2023). CDC led this effort in partnership with the Alzheimer’s Association and UsAgainstAlzheimer’s, forming a subcommittee of subject matter experts who explored various potential risk and protective factors of ADRD along three dimensions: Strength of scientific evidence, readiness for public health intervention, and potential for significant impact. This process resulted in identifying 10 risk factors meeting the criteria along the three dimensions. In 2021, after 10 years of progress on the National Plan, the NAPA Advisory Council introduced the sixth goal for the plan: Accelerate Action to Promote Healthy Aging and Reduce Risk Factors for ADRD (Goal 6; see Figure 1). This was the first and only time a new goal was added to the National Plan, reflecting the importance of addressing risk reduction and implementing interventions. Goal 6 commits federal agencies to a holistic strategy to reduce risk factors for ADRD by promoting healthy aging across all stages of life.

In October 2024, the bipartisan NAPA Reauthorization Act (Public Law No. 118-92; U.S. Congress, 2024a; see Figure 1) was signed into law, extending NAPA through 2035. This reauthorization emphasizes the importance of healthy aging and dementia risk reduction, reflecting the new sixth goal in the National Plan to promote healthy aging and reduce risk factors for ADRD. It also expanded the Advisory Council to include additional representatives from more federal agencies, ensuring a more comprehensive approach to addressing ADRD and its impact on public systems. The reauthorization of NAPA in 2024 underscores the nation's continued commitment to addressing ADRD. By extending the National Plan and embracing new goals and perspectives, the NAPA Reauthorization Act ensures that efforts to stop ADRD remain dynamic and responsive to emerging challenges. Despite this clear Congressional intent, the future of NAPA is uncertain as the Advisory Council has not been permitted to meet since January 2025 and all public members of the Advisory Council were removed from their positions in August 2025.

The RAISE Family Caregivers Act (PL 115-119; U.S. Congress, 2018a) was signed into law in 2018. The RAISE Family Caregiving Advisory Council was established to advise HHS on how to better support all family caregivers—individuals who provide essential care to loved ones with chronic illnesses, disabilities, or aging-related conditions—and not just those supporting persons living with ADRD (Administration for Community Living, 2024a). The Advisory Council develops and executes the 2022 National Strategy to Support Family Caregivers that included nearly 350 actions the federal government will take to support family caregivers and more than 150 actions that can be adopted at other levels of government and across the private sector to begin to build a system that ensures family caregivers–who provide the overwhelming majority of long-term care in the United States—have the resources they need to maintain their own health, well-being, and financial security while providing crucial support for others. The Administration for Community Living delivered a 2024 Report to Congress on Federal Implementation of the Strategy, updating actions since the National Plan release (Administration for Community Living, 2024b). Nearly all the federal actions committed to in 2022 are complete or are in progress, and federal agencies committed to almost 40 new actions since the strategy’s release. As a member of the council, the CDC considers the National Caregiving Strategy a key component of the national guidance and support available to all levels of government and the private sector to build a system that ensures family caregivers have the resources they need to support themselves and those they provide care to were aligned with the HBI and BOLD initiatives.

## 20 years of HBI: Coming full circle

The journey of the HBI and the BOLD Infrastructure for Alzheimer’s Act over the past two decades has demonstrated remarkable progress and accomplishments in addressing the challenges posed by the national public health issues of Alzheimer’s and caregiving for persons living with dementia. Through collaborative efforts among federal agencies, public health organizations, and community stakeholders, these national efforts have established a robust framework that prioritizes brain health as a critical component of public health.

The major successes of the HBI included at least the following six:

1. Establishment of national priorities through the HBI Road Map Series, Healthy People 2020 and 2030, and the addition of Goal 6 to the National Alzheimer’s Plan.
2. Establishment of national surveillance utilizing the CDC’s BRFSS and NHANES systems for cognitive decline and caregiving.
3. Legislative action for the passage and funding to establish and reauthorize BOLD to build a uniform public health infrastructure across the US, leveraging the HBI Road Map Series.
4. Establishing three PHCOE to support the nation on Dementia Risk Reduction, Early Detection and Diagnosis of Dementia, and Dementia Caregiving.
5. The awarding of two cohorts of funding to local, state, and tribal departments of health in 23 jurisdictions in the first cohort and 43 in the second cohort.
6. The support of three programs that developed and disseminated tailored resources for populations most at risk for ADRD–AI/AN adults, African American and Hispanic/Latino adults, and persons with IDD—to foster an inclusive approach to dementia care.

The year 2024 marked the reauthorizations of NAPA and the BOLD Act, leaving the US well-positioned to build on 20 years of success and to tackle the growing prevalence of Alzheimer’s disease head-on. Despite this success, the prevalence of Alzheimer's disease is rising, and so are the personal, medical, and societal burdens, highlighting the persistent unmet needs of ADRD. Health care and long-term care costs for individuals living with ADRD are substantial, as it is one of the costliest conditions for society. These health and long-term care costs for people living with ADRD are projected to reach $384 billion in 2025 and nearly $1 trillion in 2050. Nearly 12 million Americans provided unpaid care for the over 7 million people with ADRD in 2024 (Alzheimer’s Association, 2025). Additionally, in 2024, unpaid caregivers provided an estimated 19 billion hours of care, valued at nearly $413 billion.

Despite the growing burden of dementia in the US on people living with the disease and their caregivers, 2025 marked the potential end of federal support of HBI and BOLD. As part of Executive Order No. 14210 (2025), all staff with the CDC’s Alzheimer’s Disease Program were put on administrative leave in April 2025 with full termination of employment occurring in August 2025. The Alzheimer’s Disease Program was responsible for competitively funding and providing technical assistance to all of the HBI and BOLD awardees, leading the development of the HBI Road Maps, and was responsible for implementing and analyzing BRFSS and NHANES national surveillance to monitor cognitive decline and caregiving, and dissemination of that information for action.

While the unanimous bipartisan Congressional reauthorization of the BOLD Act through 2029 clearly demonstrates the intent of Congress to continue the CDC ADRD program, these actions have created enormous uncertainty around the sustainability of awardee activities and federal leadership. What will happen next is uncertain. The HBI and BOLD PHCOE recipients’ awards have been extended for 1 year, rather than terminating altogether. But there is no indication how, or even whether, there will be a re-competition for those awards to move forward with activities beyond 2026. This uncertainty puts at risk critical expertise on three crucial topics (Dementia Risk Reduction, Early Detection and Diagnosis of Dementia, and Dementia Caregiving) and four populations most at risk for ADRD (AI/AN adults, African American adults, Hispanic/Latino adults, and persons with IDD). The picture is a little better for the 43 BOLD Programs that were awarded funding for another year; however, it is unclear what will happen for the remaining two years of the 5-year award cycle. Furthermore, the BOLD Programs, with no remaining program-related staff at CDC, will receive no technical assistance or guidance on how to most effectively utilize the federal funding received. For now, the BRFSS and NHANES surveillance systems still remain and the cognitive decline and caregiving surveillance should continue. But the widespread use of these modules was largely driven by the HBI program, and its uncertain future calls into question the future of this robust data collection and utilization for state, local, and tribal action.

It is clear that the United States has made critical advancements in elevating brain health as a national public health priority during the past two decades, through the HBI and the BOLD Infrastructure for Alzheimer’s Act. Through groundbreaking surveillance, strategic funding, and the development of expert centers and inclusive programming, these initiatives laid a foundation for progress in dementia risk reduction, early detection, and caregiver support. As ADRD prevalence and associated costs escalate alongside the staggering economic and caregiving burdens, maintaining progress should be a federal priority, but it will require renewed commitment and funding from Congress. The sudden termination of CDC staff supporting HBI and BOLD programmatic activities in 2025 marks a significant and disheartening setback. The loss of leadership, infrastructure, and technical expertise, especially for communities and populations historically facing greater risks, threatens to erode the gains made and leave public health entities across the country without the resources to respond effectively. The uncertain future of the BOLD Programs and national surveillance underscores the urgent need for renewed commitment, coordination, and investment. Sustaining and building upon the legacy of the HBI and BOLD is not just a public health necessity—it is a moral imperative to support millions of Americans living with dementia and those who provide care for them. Without sustained public health infrastructure, the nation risks losing hard-won gains in addressing dementia through public health action.
